# Ciliochoroidal effusion and its association with the outcomes of micropulse transscleral laser therapy in glaucoma patients: a pilot study

**DOI:** 10.1038/s41598-022-20675-w

**Published:** 2022-09-30

**Authors:** Sunee Chansangpetch, Natnaree Taechajongjintana, Kitiya Ratanawongphaibul, Rath Itthipanichpong, Anita Manassakorn, Visanee Tantisevi, Prin Rojanapongpun, Shan C. Lin

**Affiliations:** 1 Center of Excellence in Glaucoma, Department of Ophthalmology, Faculty of Medicine, Chulalongkorn University, Bangkok, Thailand; 2 King Chulalongkorn Memorial Hospital, Thai Red Cross Society, Bangkok, Thailand; 3 Glaucoma Center of San Francisco, San Francisco, CA USA

**Keywords:** Eye diseases, Translational research

## Abstract

We investigate the development of ciliochoroidal effusion following micropulse transscleral laser therapy (MPTLT) and evaluate the relationship between the early postoperative ciliochoroidal effusion (ECE) and short-term treatment outcomes. Glaucoma patients who underwent MPTLT were assessed for ciliochoroidal effusion by anterior segment optical coherence tomography (AS-OCT) at postoperative 1, 4, 12 weeks. The subjects were classified based on AS-OCT findings at postoperative 1 week into eyes with and without ECE. The absolute intraocular pressure (IOP), IOP reduction and number of antiglaucoma medications were compared between eyes with and without ECE. A total of 50 eyes were included, of which 23 (46%) developed ciliochoroidal effusion at postoperative 1 week. Almost all effusion resolved at 4 weeks. At 12 weeks, the mean IOP (SD) significantly decreased from 28.5 (12.8) mmHg to 17.8 (10.5) mmHg (p < 0.001), and the mean number of medications (SD) decreased from 4.1 (0.9) to 3.3 (1.1) (p < 0.001). Eyes with ECE had significantly greater IOP reduction (p = 0.009) and lower absolute IOP (p = 0.008) at the 4-week visit. There was no significant difference in number of medications between the groups. In conclusion, ciliochoroidal effusion was commonly observed following MPTLT. Eyes with ECE had overall greater IOP reduction during early post-operation.

## Introduction

Glaucoma is the leading cause of blindness worldwide^1,2^. The aim of glaucoma treatment is to lower intraocular pressure (IOP) through various medications, laser treatment, surgeries, and cyclodestructive procedures.

Micropulse transscleral laser therapy (MPTLT) is a novel alternative glaucoma laser treatment that uses a short repetitive burst of laser energy in the ciliary body and pars plana region. This method is believed to incur less destruction of collateral tissue. Previous studies have demonstrated that MPTLT is effective in reducing IOP with lower complication rates compared to traditional continuous-wave transscleral cyclophotocoagulation^3–5^. Unlike conventional cyclodestructive procedures, in which the onset of IOP lowering occurs at 4–6 weeks post-treatment^6^, literature shows that MPTLT can reduce IOP as early as the first week post-operation^3,7–19^. The exact IOP lowering mechanism of MPTLT remains unclear. Some possible mechanisms for IOP reduction that have been proposed include the following: (1) subthreshold cell damage and inflammation of the ciliary body, which leads to the reduction of aqueous humor production, (2) an increase in uveoscleral outflow, and (3) a pilocarpine-like effect resulting from the contraction of longitudinal fibers of the ciliary muscle^5,20,21^.

Studies have found that an increase in uveoscleral outflow can occur during the early postoperative period in several glaucoma procedures, such as trabeculectomy^22–24^, deep sclerectomy^25,26^, trabeculotomy^27,28^, and microstent implantation^29^. The presumptive enhanced uveoscleral outflow was transient and corresponded with the development of ciliochoroidal effusion^27,28^. No previous study has investigated whether MPTLT can produce a similar effect.

Anterior segment optical coherence tomography (AS-OCT) is an imaging modality used to investigate anterior segment morphology^30^. This imaging method can also be used to detect changes in the supraciliary region^24,27,31^. Herein, we describe the incidence of ciliochoroidal effusion following MPTLT using AS-OCT and evaluate the relationship between the effusion and treatment outcomes during a 3-month follow-up period.

## Methods

This was a prospective study of all consecutive glaucoma patients who underwent MPTLT (IRIDEX Cyclo G6^®^ Glaucoma Laser System with MicroPulse P3^®^ Glaucoma Device, IRIDEX Corporation, Mountain View, CA) for uncontrolled glaucoma despite maximum tolerated medical therapy at the glaucoma outpatient department, Faculty of Medicine, Chulalongkorn University between January 2020 and January 2021. Patients with difficulty performing AS-OCT, such as those with neck and back problems, were excluded from the study. Patients with conjunctival pathologies, orbital or eyelid anomalies that limit visualization of the supraciliary region on AS-OCT, or a prior history or evidence of choroidal abnormalities that could interfere with imaging were also excluded. This study was approved by the Institutional Review Board of the Faculty of Medicine, Chulalongkorn University, Bangkok, Thailand. This study was conducted in accordance with the tenets of the Declaration of Helsinki. Written informed consent was obtained from all the participants.

The following baseline ophthalmic information were recorded prior to the procedure: glaucoma diagnosis, antiglaucoma medications, previous glaucoma surgery or laser treatment, preoperative visual acuity, lens status, cup-to-disc ratio, and IOP. Patients were monitored on the day after the operation and at 1, 4, and 12 weeks postoperatively. At each visit, the number of glaucoma medications taken, postoperative visual acuity, and IOP were recorded. AS-OCT was also performed at baseline and at postoperative 1, 4, and 12 weeks.

### Operative procedures

Glaucoma specialists performed the MPTLT using an IRIDEX Cyclo G6^®^ Glaucoma Laser System with a MicroPulse P3^®^ Glaucoma device using standardized protocols and techniques. The procedure was performed under local anesthetic retrobulbar or peribulbar injection with 2–5 ml of 2% lidocaine hydrochloride along with topical 2% xylocaine gel. The MPTLT was conducted using a standardized power of 2000 mW and a duty cycle of 31.3%. The MicroPulse^®^ P3 glaucoma laser probe was placed adjacent to the limbus with steady pressure, sweeping along the superior and inferior halves of the sclera in a continuous motion, avoiding the 3 and 9 o’clock positions and any area of thin sclera. There were two presets of variable duration in this study: (1) 90 s each half (for a total of 180 s) for first-time MPTLT patients, and (2) 140 s each half (for a total of 280 s) for repeat MPTLT patients.

Postoperative medications included topical prednisolone acetate 1% four times daily, with dosage gradually reduced depending on the degree of inflammation, while maintaining previously prescribed IOP-lowering medications. Antiglaucoma medical treatment was adjusted at each follow-up visit and reduced when possible, according to the target IOP in a stepwise approach, with oral carbonic anhydrase inhibitors being the first medication to be stopped. Other topical medications were reduced in the following order: prostaglandin analogs, alpha agonists, carbonic anhydrase inhibitors, and beta-blockers. Each medication was discontinued one at a time unless the IOP was less than 7 mmHg, and the adjustment was at the discretion of the clinicians.

### Image acquisition

**Figure 1:**
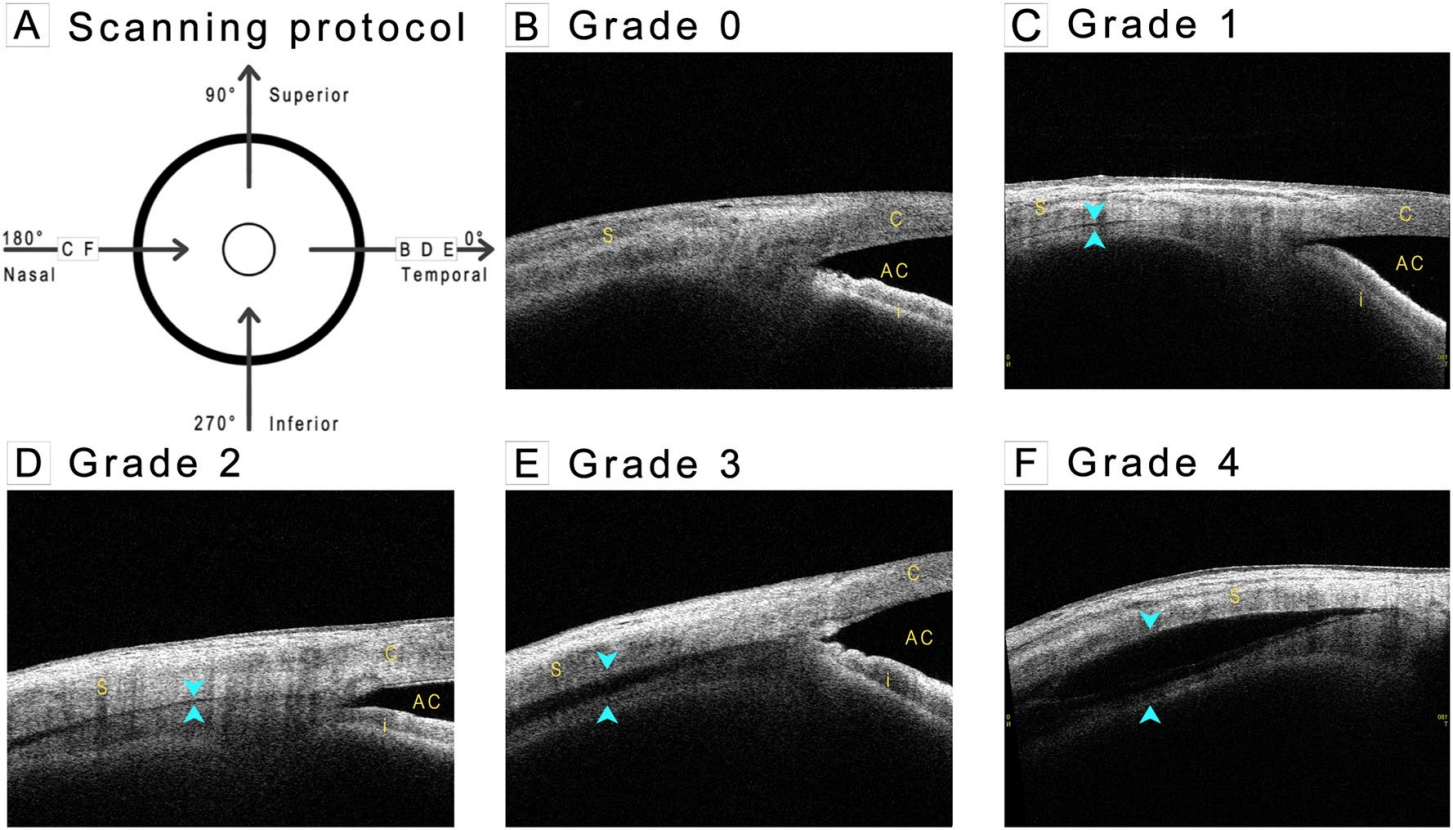
Anterior segment optical coherence tomography scanning protocol and representative images of hyporeflective signal grading of supraciliary change. Arrowhead indicates hyporeflective signal at supraciliary region. *S* scleral, *C* cornea, *AC* anterior chamber, *I* iris.

An experienced technician performed AS-OCT (CASIA2; Tomey Corporation, Nagoya, Japan) in a standardized dark environment to assess the suprachoroidal space. Patients were asked to look at four cardinal directions (i.e., up, down, medial, and lateral) successively using internal fixating lights. The scans were subsequently performed over the sclera at four locations (3, 6, 9, and 12 o’clock), resulting in a set of four scans per patient. The scans were acquired using the Angle HD preset, which comprises 64 raster scans of 800 A-scans per line and a scanning dimension of L8 mm × W4 mm × D11 mm. To standardize the location of the scan, the scan window was centered at the limbus, with the length of the rectangle perpendicular to the limbal line (Fig. 1A).

### Image analysis

The volumetric scans of each location were evaluated for supraciliary change by determining hyporeflective signals between the ciliary body and sclera and classified based on its continuity and height into a 5-scale height grading (grades 0 to 4) as follows:

Grade 0: No visualized hyporeflective signal

Grade 1: Hyporeflective slit in localized, discontinuous, or skip patterns

Grade 2: Continuous hyporeflective slit

Grade 3: Continuous hyporeflective band of less than half of the maximum scleral thickness

Grade 4: Continuous hyporeflective band of at least half of the maximum scleral thickness.

All cross-sectional images from volumetric scans at each location were reviewed for the presence of hyporeflective signals. If the hyporeflective signal did not have a skip pattern, it was classified based on its height relative to the scleral thickness at the cross-sectional image that had the most prominent effusion. Figure 1B–F shows representative images of grades 0–4. All scans were graded by a single fellowship-trained glaucoma specialist (NT) who was blinded to the patients’ clinical data.

### Outcomes

For each eye, the hyporeflective supraciliary change was reported in terms of the following: (1) height grading–summation of the grading (0 to 4) from four locations, ranging from 0 (all locations were grade 0) to 16 (all locations were grade 4), and (2) extent, number of involved locations (demonstrating any grade of supraciliary change), ranging from 0 (none) to 4 (all 4 locations). Ciliochoroidal effusion was defined as hyporeflective signals of at least grade 2 in at least one location.

The subjects were classified based on the presence of ciliochoroidal effusion at postoperative 1 week into eyes with early ciliochoroidal effusion (ECE) and eyes without early ciliochoroidal effusion (no ECE). The MPTLT outcomes included (1) percentage of IOP reduction (100* [IOP_baseline_ − IOP_follow-up_]/IOP_baseline_), (2) absolute IOP, and (3) number of antiglaucoma medications.

### Statistical analysis

A paired t-test was used to compare MPTLT outcomes between the baseline and 12 weeks. Baseline characteristics between the ECE and no ECE groups were compared using t-tests, chi-square tests, and Fisher’s exact tests, depending on the data type. The MPTLT outcomes at each follow-up time point were compared between the two groups using the t-test and multivariable linear regression models with adjustment for baseline IOP and previous glaucoma interventions. Mixed effect linear regression analyses were conducted to assess the overall relationship between ECE and IOP reduction during the 3-month course of follow-up. The model was built to account for previous glaucoma interventions, the number of medications, follow-up time, and the interaction between the number of medications and follow-up time. All analyses were performed using Stata 16.0 (Stata Corp., College Station, TX, USA). Statistical significance was set at p value < 0.05.

## Results

Fifty eyes of 50 patients were included in this study. The mean IOP (SD) at baseline was 28.5 (12.8) mmHg, which significantly decreased to 17.8 (10.5) mmHg at 12 weeks (p < 0.001). The mean number of preoperative medications was 4.1 (0.9) and significantly decreased to 3.3 (1.1) at 12 weeks (p < 0.001). The IOP reached a nadir of 12.3 (9.6) mmHg at postoperative 1-week. The mean 12-week IOP reduction rate was 33%.

All the subjects had normal baseline AS-OCT findings. At postoperative 1-week, hyporeflective signals in the supraciliary region were detected in 38 eyes (76%). Among them, 23 (46%) eyes met the criteria for ciliochoroidal effusion. The median (interquartile range [IQR]) height grading was 3 (range, 1–6), and the median (IQR) extent was 2 (1–3). The effusion substantially decreased at the 4-week follow-up. There was only one eye in which effusion persisted at four and 12 weeks. None of the patients had visible choroidal detachment, as assessed by indirect ophthalmoscopy examination. The progression of the hyporeflective signal in terms of height and extent during the 12-week study period is shown in Table 1. The distribution of the supraciliary hyporeflective signals per location is shown in Supplementary Table 1.

**Table 1:** Hyporeflective supraciliary change.

	Week 1	Month 1	Month 3
Height (height grading)	3.80 ± 3.65	0.33 ± 0.80	0.55 ± 2.43
Extent (number of locations)	2.16 ± 1.49	0.31 ± 0.70	0.24 ± 0.83
Eyes with any hyporeflective signal	38 (76)	9 (18)	3 (6)
Eyes with ciliochoroidal effusion	23 (46)	1 (2)	1 (2)

Data present in mean ± standard deviation and N (%).

The demographic data and baseline characteristics of all eyes and eyes with and without ECE are shown in Table 2. The baseline characteristics of patients with and without ECE were comparable. Fisher’s exact test compared the history of filtering surgery between the two groups showed that those without ECE tended to have higher rates of history of filtering surgery, but the p-value did not reach a statistically significant level after Bonferroni correction for multiple testing. The diagnostic subtypes of secondary glaucoma are shown in Supplementary Table 2.

**Table 2:** Demographic data and baseline characteristics.

	All	No ECE	ECE	P value
N	50	27 (54.0)	23 (46.0)	
Age (years)	59.7 ± 16.7	59.3 ± 17.9	60.1 ± 15.5	0.88
**Gender**				0.30
Male	30 (60.0)	18 (66.7)	12 (52.2)	
Female	20 (40.0)	9 (33.3)	11 (47.8)	
**Diagnosis**				0.62*
POAG	10 (20.0)	7 (25.9)	3 (13.0)	
PACG	3 (6.0)	2 (7.4)	1 (4.3)	
Secondary glaucoma	34 (68.0)	17 (63.0)	17 (73.9)	
Childhood glaucoma	3 (6.0)	1 (3.7)	2 (8.7)	
Diabetic mellitus	8 (16.0)	6 (22.0)	2 (8.7)	0.26*
Hypertension	9 (18.0)	7 (25.9)	2 (8.7)	0.15*
Dyslipidemia	14 (28.0)	7 (25.9)	7 (30.4)	0.72
**Lens status**				0.74*
Phakic	16 (32.0)	10 (37.0)	6 (26.1)	
Pseudophakic	26 (52.0)	13 (48.1)	13 (56.5)	
Aphakic	8 (16.0)	4 (14.8)	4 (17.4)	
**First/repeated treatment**				0.99
First	37 (74.0)	20 (74.1)	17 (73.9)	
Repeated	13 (26.0)	7 (25.9)	6 (26.1)	
**Previous intervention**				0.01*
None	25 (50.0)	9 (33.3)	16 (69.6)	
Filtering surgery	20 (40.0)	15 (55.6)	5 (21.7)	
Cyclophotocoagulation	4 (8.0)	3 (11.1)	1 (4.3)	
Canal-based surgery	1 (2.0)	0 (0.0)	1 (4.3)	
Visual acuity (logMAR)	1.83 ± 0.94	1.93 ± 0.92	1.71 ± 0.98	0.44
Cup-to-disc ratio^†^	0.87 ± 0.12	0.88 ± 0.11	0.85 ± 0.14	0.43
Intraocular pressure (mmHg)	28.7 ± 12.8	26.9 ± 13.9	30.3 ± 11.4	0.35
Number of glaucoma medications	4.1 ± 0.9	4.2 ± 0.7	4.0 ± 1.0	0.72

Data present in N (%) and mean ± standard deviation.

Significant p value after Bonferroni correction = 0.004.

*ECE* early ciliochoroidal effusion, *POAG* primary open angle glaucoma, *PACG* primary angle closure glaucoma.

*Fisher’s exact test.

^†^Visible cup-to-disc ratio in 40 patients (no ECE = 22 and ECE = 18).

**Figure 2:**
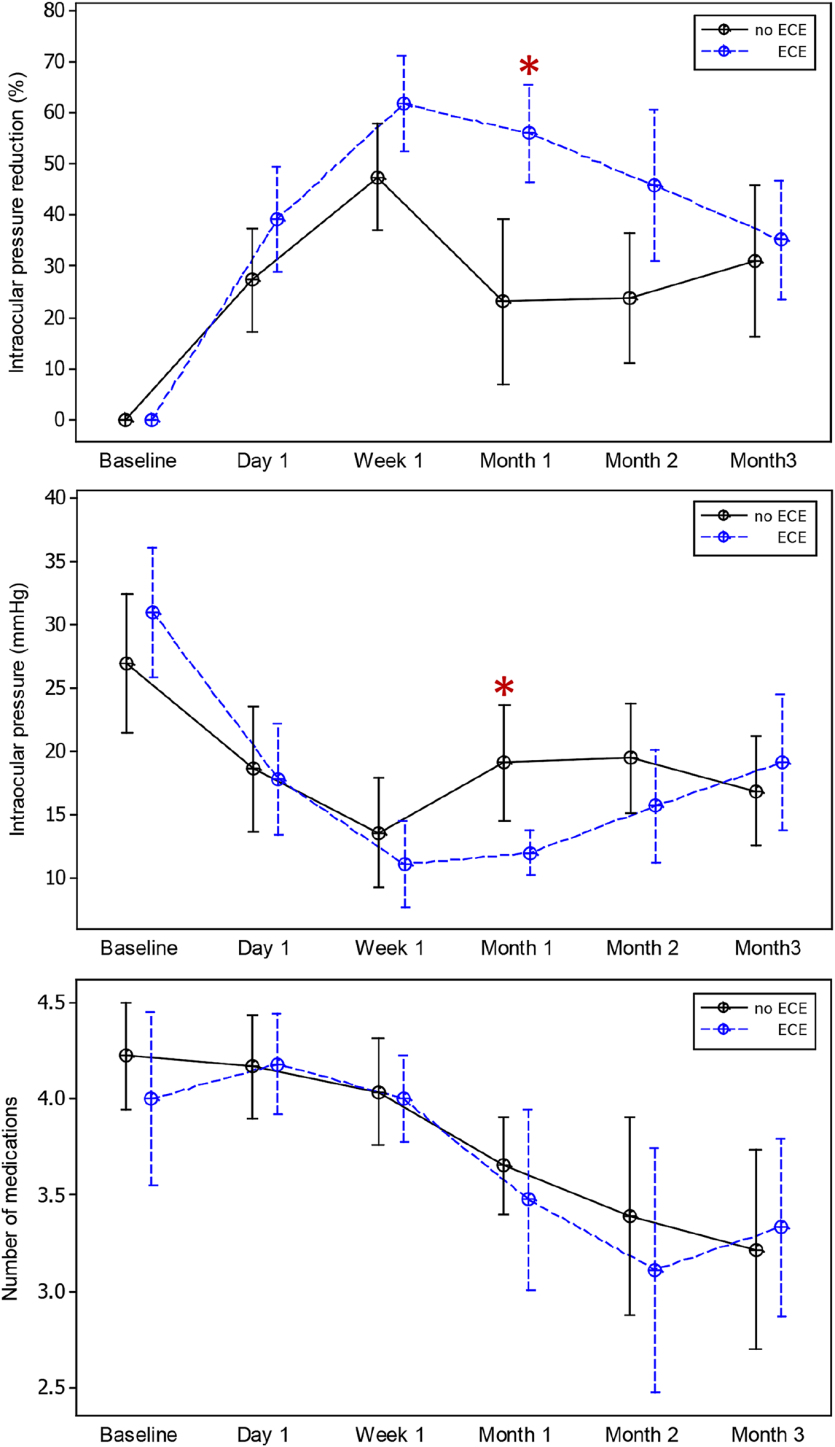
Interval plots of micropulse transscleral laser therapy outcomes during 12 weeks. (Top) intraocular pressure reduction, (Middle) absolute intraocular pressure, (bottom) number of glaucoma medications. Asterisk indicates statistically significant difference.

The difference in IOP reduction between the two groups showed statistical significance at one month, with greater IOP reduction in the ECE group (mean difference −32.8%, 95% CI −52.3 to −13.3, p = 0.001). Multivariable regression analysis adjusted for preoperative IOP and previous glaucoma surgery revealed similar results (β −30.7, 95% CI –53.42 to −8.03, p = 0.009). For absolute IOP, the analysis showed significantly lower absolute IOP at one month in the ECE group (mean difference 5.87 mmHg, 95% CI 0.29 to 11.46 mmHg, p = 0.040). The difference remained significant after controlling for covariates (β = 8.09, 95% CI 2.24 to 13.93, p = 0.008). At three months postoperatively, the differences between both groups were not statistically significant (mean IOP reduction – No ECE 31.09% versus ECE 35.15%, p = 0.61; mean absolute IOP – No ECE 16.83 mmHg versus ECE 18.95 mmHg, p = 0.91). There was no significant difference in the number of medications between the two groups at any time point (all p > 0.05). IOP reduction, absolute IOP, and number of medications at each time point are shown in Fig. 2 and Supplementary Table 3.

The mixed model regression revealed that the presence of ECE was significantly associated with greater IOP reduction (β = 12.3, 95%CI 2.85 to 21.85, p = 0.011) during the 3-month period after treatment.

The amount of effusion post-MPTLT in terms of height grading and extent did not show any significant association with IOP reduction at one week, one month, or three months (all p > 0.10). The analysis with a mixed-effects model also did not reveal an association between the amount of effusion at 1-week post-treatment and the overall percentage of IOP reduction during the 3-month period (all p > 0.20).

## Discussion

In this prospective descriptive study, we found hyporeflective supraciliary changes following MPTLT in glaucoma patients. Ciliochoroidal effusion was observed by AS-OCT beginning at one week post-operation examination. In some cases, this persisted for 3 months. MPTLT treatment was associated with a significant IOP reduction, and the amount of reduction was correlated with the presence of ECE.

Our study demonstrated a 12-week IOP reduction of 33%, which was greater than the 25.9% reduction reported by Kaba et al. The greater IOP reduction could be explained by the higher power used in our protocol^4^. A study from Radhakrishnan et al., in which their laser settings were roughly equivalent to ours, found a comparable IOP reduction of 28%^32^. Zaarour and colleagues also reported an average of 29.2% IOP reduction at 3 months post-operation using a similar standardized MPTLT protocol to our study^9^.

To our knowledge, this is the first study to describe ciliochoroidal effusion using AS-OCT and its relationship with IOP reduction after MPTLT. Several potential mechanisms may explain the development of ciliochoroidal effusion.

First, ciliochoroidal effusion could be the result of a decrease in IOP following MPTLT. In the hypotonous state, low IOP and accumulation of serum proteins in the suprachoroidal space can cause the disruption of hydrostatic and oncotic pressures^33–36^. An imbalance of these forces in the suprachoroidal space leads to the development of choroidal effusion which usually occurs at IOPs below that of the episcleral venous pressure^37^. The hypothesis is ascertained by studies in trabeculectomy which showed choroidal detachment occurred at a mean IOP of 5.5 to 6.1 mmHg^38,39^. However, our study found that ciliochoroidal effusion developed at a higher IOP level. The reduction in IOP may not completely explain the development of effusion.

A recent study by Johnstone et al. demonstrated ciliary muscle shortening during MPTLT in an enucleated monkey eye (Johnstone MA, et al. IOVS 2017;58:ARVO E-Abstract 3468). The study suggested that contraction of the ciliary muscle could change the trabecular meshwork and conventional outflow pathway shape, the mechanism of which is similar to that of pilocarpine’s IOP-lowering effect. Interestingly, pilocarpine has been shown to be associated with ciliary edema and uveal effusion^40,41^. Although the exact pathogenesis underlying fluid accumulation from pilocarpine is not fully understood, the possible association between the ciliary muscle contraction effect and effusion could not be ruled out.

Another explanation for the effusion formation may be the proposed IOP-lowering mechanism of MPTLT pertaining to enhancing aqueous outflow through a uveoscleral pathway^13^. Although there is no conclusive evidence that imaging-detectable ciliochoroidal effusion is a sign of increased uveoscleral drainage, there are few reports that support this presumption^22,23^. Ito et al. observed supraciliochoroidal fluid in post-trabeculectomy eyes that had an IOP of less than 15 mmHg without functioning blebs^22^. The study suggested that increased uveoscleral outflow is a possible mechanism for the occurrence and that supraciliochoroidal fluid could be indicative of an increased uveoscleral outflow.

**Figure 3:**
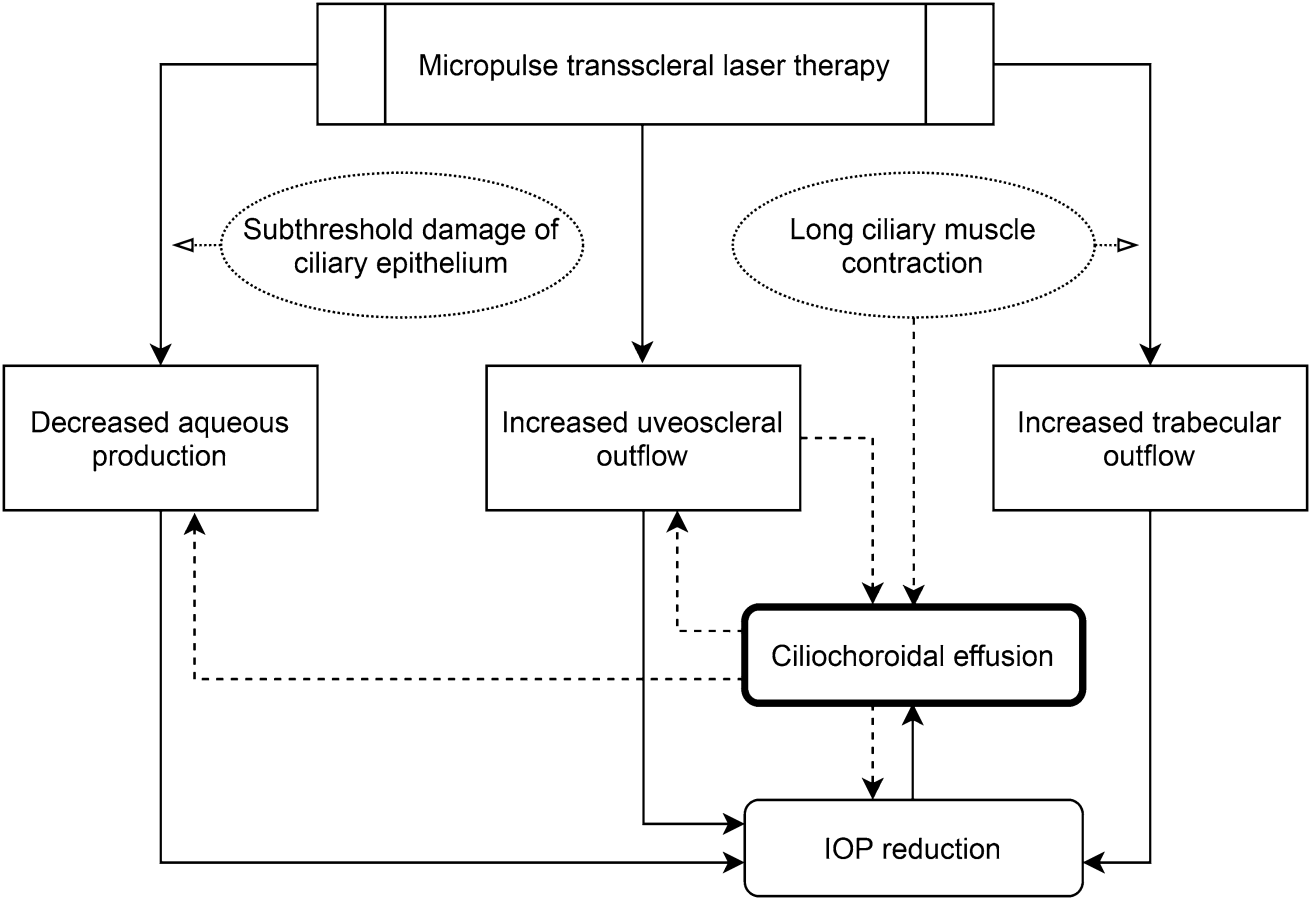
Possible mechanisms for intraocular pressure reduction and ciliochoroidal effusion in micropulse transscleral laser therapy. Dashed line indicates proposed mechanisms of ciliochoroidal effusion. *IOP* intraocular pressure.

The specific pathophysiology of ciliochoroidal effusion after MPTLT cannot be fully explained; however, a potential explanation could be a combination of the three mechanisms mentioned above. Our findings suggest that MPTLT can increase uveoscleral drainage. Nevertheless, this proposed mechanism warrants further studies to measure aqueous drainage via the unconventional pathway, for example, by direct measurement using tracers (tracer-based methods) or indirect methods to estimate unconventional outflow from the difference between aqueous humor production and outflow through the conventional pathway^42^. The proposed mechanism of ciliochoroidal effusion and its relation to the IOP-lowering effect is outlined in Fig. 3.

Notably, the formation of ciliochoroidal effusion per se can directly reduce IOP. An experimental ciliochoroidal detachment has been shown to lower IOP, presumably due to reduced aqueous production and enhanced uveoscleral drainage^43^. This is in line with our observation of a slight IOP rebound once the effusion resolved. However, IOP was mostly maintained at a low level after closure of the supraciliary space, indicating that the principal mechanism for sustained IOP lowering was likely due to other mechanisms, rather than the transient effect from early ciliochoroidal detachment.

Whether ciliochoroidal effusion is caused by increased uveoscleral outflow or effusion causes increased uveoscleral outflow, transient ciliochoroidal effusion may be a major mechanism of early IOP reduction. Data from Radhakrishnan et al. and Kaba et al. indicated that the peak IOP reduction was at 1 week post-operation^4,32^. After the initial IOP drop phase, IOP increased to reach a plateau at approximately 1 to 3 months^7^. These findings were consistent with the data obtained in our study. ECE could explain the initial IOP drop phase within the first month of MPTLT as well as the faster onset of IOP reduction compared to traditional cyclophotocoagulation, which typically takes 4–6 weeks to begin. Our findings indicate that patients who developed ECE were likely to have a longer initial IOP drop phase. While the no ECE group had IOP that reached a plateau at 1 month, the IOP in the ECE group gradually increased and became comparable to the no ECE group at two to three months post-operation. Reducing or discontinuing antiglaucoma medications too early after a good initial response could result in a greater IOP rebound afterwards. In addition, as shown in Fig. 2, an increase in IOP in the ECE group at postoperative 3rd month could partly be a consequence of tapering medications during the prior low-IOP visits at 1 and 2 months. An increase in IOP from reducing medications could be a critical issue for patients with advanced glaucoma. Supraciliary assessment could be an adjunctive tool for postoperative monitoring, especially during medication adjustment, among high-risk individuals who may have early IOP responses to laser treatment. Overall, the eyes with ECE had better initial MPTLT outcomes. Nevertheless, long-term follow-up is needed to further confirm the treatment outcomes.

This study has several limitations. First, the sample size was limited and included only an Asian population, with a relatively short follow-up time. To make a more generalized statement, a larger and more ethnically diverse sample size and longer follow-up period are needed. Additionally, we only assessed supraciliary changes at the four locations. More detailed investigations, such as circumferential assessments, will provide more information. Lastly, the exact onset of suprachoroidal fluid presence could not be identified because AS-OCT scanning was performed at 1 week post-operation. We were unable to conduct AS-OCT imaging sooner because of patient discomfort after the operation. Moreover, image quality could be limited by some of the early postoperative conditions, such as conjunctival chemosis and subconjunctival hemorrhage.

In conclusion, MPTLT is an effective treatment for lowering IOP and helps to reduce the number of IOP-lowering medications. Ciliochoroidal effusion was observed after treatment in nearly half of the patients. The presence of effusion may indicate a relationship between the MPTLT and uveoscleral drainage. Eyes with effusion after 1 week had an overall greater IOP reduction during the early postoperative period.

## Supplementary Information

Supplementary Tables.

## Data Availability

The datasets generated during the current study are available from the corresponding author upon request.
