# Supplementary material for: Ciliochoroidal effusion and its association with the outcomes of micropulse transscleral laser therapy in glaucoma patients: a pilot study
Source: Sci Rep. 2022 Sep 30;12:16403. doi: 10.1038/s41598-022-20675-w (PMC9525712; doi:10.1038/s41598-022-20675-w)
Supplement: Supplementary file 1 — Supplementary Tables. [file 41598_2022_20675_MOESM1_ESM.pdf]

Supplement table 1 Distribution of supraciliary hyporeflective signal per location

	Superior	Nasal	Inferior	Temporal
week 1	24	32	23	29
month 1	2	7	1	4
month 3	1	2	2	2

Data present in N

Supplement table 2 Diagnosis subtypes of secondary glaucoma

	No ECE	ECE
Pseudoexfoliation	0	1
Post corneal transplantation	4	7
ICE syndrome	1	0
Neovascular glaucoma	6	2
Uveitis glaucoma (without NVG)	1	0
Trauma	0	3
Associated with cataract complication (e.g. aphakic glaucoma)	3	2
Associated with retina surgery	2	1
Craniofacial anomalies	0	1
Total	17	17

ECE early ciliochoroidal effusion

Fisher's exact test P value =0.22

Supplement table 3 Intraocular pressure reduction, absolute intraocular pressure, and number of medications at each time point

	All	No ECE	ECE	Mean difference (95% CI)	P value 1	P value 2
IOP reduction (%)						
• day1	33.02 (24.02)	27.37 (24.03)	39.17 (22.97)	-11.80 (-25.80 to 2.19)	0.096	0.337
• week1	54.00 (25.22)	47.44 (26.54)	61.70 (21.67)	-14.26 (-28.20 to -0.32)	**0.045**	0.149
• month1	37.79 (36.50)	23.14 (39.97)	55.93 (21.08)	-32.79 (-52.25 to -13.33)	**0.001**	**0.009**
• month2	33.47 (31.17)	23.80 (29.29)	45.81 (29.79)	-22.01 (-40.79 to -3.22)	**0.023**	0.273
• month3	33.03 (30.01)	31.09 (34.09)	35.15 (25.47)	-4.07 (-22.52 to 14.39)	0.659	0.610
Absolute IOP (mmHg)						
• baseline	28.46 (12.82)	26.91 (13.93)	30.28 (11.41)	-3.38 (-10.70 to 3.95)	0.359	-
• day1	18.23 (10.52)	18.58 (11.64)	17.84 (9.40)	0.74 (-5.58 to 7.07)	0.814	0.293
• week1	12.33 (9.62)	13.40 (10.88)	10.91 (7.91)	2.62 (-2.87 to 8.12)	0.342	0.213
• month1	16.11 (9.80)	18.73 (11.03)	12.86 (6.98)	5.87 (0.29 to 11.46)	**0.040**	**0.008**
• month2	17.51 (9.24)	18.96 (9.77)	15.67 (8.43)	3.29 (-2.57 to 9.15)	0.263	0.252
• month3	17.84 (10.52)	16.83 (9.98)	18.95 (11.23)	-2.13 (-8.58 to 4.33)	0.510	0.911
P value 3	**<0.001**	**<0.001**	**<0.001**			
Number of medications						
• baseline	4.12 (0.87)	4.22 (0.70)	4.00 (1.04)	0.22 (-0.28 to 0.72)	0.375	-
• day1	4.17 (0.61)	4.17 (0.64)	4.18 (0.59)	-0.02 (-0.38 to 0.35)	0.934	0.846
• week1	4.02 (0.62)	4.04 (0.71)	4.00 (0.52)	0.04 (-0.32 to 0.40)	0.836	0.953
• month1	3.57 (0.83)	3.65 (0.63)	3.48 (1.03)	0.18 (-0.31 to 0.67)	0.470	0.678
• month2	3.27 (1.23)	3.39 (1.20)	3.11 (1.28)	0.28 (-0.50 to 1.06)	0.474	0.460
• month3	3.27 (1.11)	3.22 (1.20)	3.33 (1.02)	-0.12 (-0.80 to 0.57)	0.733	0.948
P value 3	**<0.001**	**0.002**	**0.001**			

P value 1 t test
P value 2 linear regression with the adjustment of baseline IOP and previous glaucoma surgery
P value 3 paired t test

ECE early ciliochoroidal effusion, IOP intraocular pressure
